# Research on Decision-Making of Hybrid Dominant Closed-Loop Supply Chain considering Different Logistics Modes

**DOI:** 10.1155/2022/9889292

**Published:** 2022-10-10

**Authors:** Wanxian Wu, Lin Miao

**Affiliations:** Business School of Liming Vocational University, Quanzhou 362000, China

## Abstract

Based on the mode of manufacturers dominating in the forward supply chain and retailers dominating in the backward supply chain, a hybrid leading closed-loop supply chain structure with different two-way dominance is constructed to study the advantages and disadvantages of supply chain decision-making under logistics self-supporting and logistics outsourcing modes. Stackelberg game theory is used to solve the equilibrium solution and optimal profit of the forward and backward supply chains under different logistics modes. On this basis, the profits of manufacturers and retailers in the forward and backward closed-loop supply chains are compared, and finally, the conclusion is verified by numerical examples. The influence of different logistics modes on pricing decisions of manufacturers and retailers is studied based on the dual-agent hybrid dominant supply chain model, and then, the logistics mode selection of manufacturers and retailers under different conditions of forward dominant body and backward dominant body.

## 1. Introduction

When the whole world begins to gradually enter the postindustrial era, it is urgent to reduce environmental pollution and improve the efficiency of resource utilization. Therefore, the government proposes to strongly support enterprises to recycle waste products for remanufacturing. Compared with traditional supply chain systems, the recycling and remanufacturing closed-loop supply chain has a complex structure, and dominant enterprises, recycling channels, and logistics services have a cross-impact on the selection of closed-loop supply chain mode. Based on the reproduction capacity or recycling capacity, the closed-loop supply chain is mostly dominated by manufacturers or retailers [1]. The closed-loop supply chain dominated by different players needs support from different recycling channels and decides whether to adopt logistics outsourcing [2]. Savaskan and Wassenhove [3] studied the closed-loop supply chain with different recycling channels established by manufacturers, sellers, and third parties in charge of recycling, respectively, and the results showed that sellers in charge of recycling were more effective than manufacturers and third parties. Han [4] and Gao et al. [5] studied three recovery channel strategies of retailer recovery, manufacturer recovery, and third-party recovery in the closed-loop supply chain dominated by sellers. Gong [6] designed four supply chain modes based on the cross-combination of manufacturer- or seller-dominant mode and recovery mode and then analyzed the relationship between sales price, sales volume, recovery rate, and profit under different modes to determine the stability of the four supply chains. Meanwhile, the above dominant modes combine logistics self-supporting or outsourcing to form a new combination, and its stability is evaluated through the total profit of the supply chain [7]. Zhang et al. [8] proposed that enterprises in the forward and backward processes of the closed-loop supply chain had different concerns, so the dominant enterprises in the supply chain should choose different logistics strategies to achieve the stability of the supply chain.

The cross-combination of different dominant modes, recovery modes, and logistics strategies forms closed-loop supply chains with different structures. The structure of the closed-loop supply chain affects supply chain pricing, main income (profit distribution), and supply chain stability and ultimately influences the choice of supply chain model. Karakayali et al. [9] studied the pricing service decision of the backward supply chain from the perspective of the power structure of different channels. Ma et al. [10] studied the two closed-loop supply chains with or without government subsidies and explored the benefits that consumers obtained from them. Gao et al. [11] studied the product pricing and member enterprises' profit distribution in the closed-loop supply chain under three situations: manufacturers' dominance, retailers' dominance, and manufacturers and retailers' rights, and believed that retailers' profits were the largest rights when they dominated, and under the condition of equal rights, this was most favorable for consumers, and at this time, the product sales price was the lowest. Liu and Liu [12] analyzed the dual-channel closed-loop supply chain system in which the original manufacturers and the third-party remanufacturing coexisted, analyzed the differential pricing methods of new products and remanufactured products by using the game model, and then obtained the best pricing strategies under different modes. Sun et al. [13] constructed a closed-loop supply chain structure of three recycling channels based on the influence of sales quantity and recycling price. Based on the closed-loop supply chain structure with double sales and double recovery as two competitive channels, Lin and Cao [14] explored the pricing strategy model of the closed-loop supply chain with manufacturers and retailers as the dominant players. Xie [15] believed that revenue sharing contract could provide support for supply chain cooperation and proposed that forward sales revenue and backward recovery revenue should be distributed simultaneously based on contract theory, which would help maximize the profits of both in the closed-loop supply chain system cooperated by a single manufacturer and single retailer. Ding and Ma [16] conducted a comparative analysis on pricing decisions and benefits of the two closed-loop supply chain models in which suppliers chose to participate in parts recycling and remanufacturing under the situation of supplier dominance. Xu et al. [17] analyzed the dual-channel supply chain model and obtained several management suggestions on delivery lead time decision-making. Matsui [18] used an observable delay game framework in noncooperative game theory to analyze pricing decisions in dual-channel supply chains. By modeling the demand function of the two channels as a linear function of the retail price and direct price, when manufacturers should set their direct price and wholesale price in the dual-channel supply chain was revealed. Batarfi et al. [19] extended the two-channel supply chain to a setup where standard and customized products were sold through retailers and online channels, respectively. The paper took demand as a linear function of sales price, quoted delivery time, and product difference and determined the best business strategy to maximize the total profit of the system. Besides, they also studied the impact of dual-channel strategy on supply chain performance when inventory costs were incorporated into the system. Meng et al. [20] used Stackelberg game theory to establish a dual-channel green supply chain model of consumers' green preference and channel preference. The research results show that the higher the consumer's green preference or the lower the offline channel preference, the greater the demand for green products. Zhang et al. [21] studied the dynamic pricing strategy problem of a dual-channel supply chain composed of manufacturers and retailers under consumers' green preferences. The research structure shows that the market demand is concave with the selling price, and when the selling price is equal to the reference price, the market demand reaches the maximum value, so the manufacturer should strategically decide to change the price in real time according to the previous period price. Considering consumer product differentiation preferences, Zhang and Zheng [22] studied the company's optimal customization strategy and commodity diversification pricing decisions under both online and offline channels, as well as the impact of customization strategies on company pricing decisions, profits, and consumer welfare. It is shown that, under unified pricing, the only factor affecting the customization strategy of a single-channel company is its cost efficiency, and the customization strategy is likely to play a strategic role in correctly guiding consumers' “total traffic” level.

In conclusion, the closed-loop supply chain structure or mode is influenced by the dominant enterprises, recycling channels, logistics services, and other factors [23–25], and the closed-loop supply chain structure affects the dominant enterprises' choice of closed-loop supply mode based on product pricing, enterprise benefits, and other factors. These conclusions have reached a consensus in the academic community, which is also the basis of this study. Existing studies may have the following shortcomings: (1) most of them focus on the closed-loop supply chain structure of single logistics service of logistics self-supporting or logistics outsourcing, and there are few studies on the selection and comparison of logistics self-supporting and logistics outsourcing services; (2) most of the studies on the closed-loop supply chain considering logistics services do not reflect the impact of logistics cost on the selection of recovery channel and supply chain mode; (3) existing studies are mainly manufacturer-dominant or retailer-dominant closed-loop supply chain systems, without distinguishing the leading enterprises of forward and backward supply chain systems, but in the actual environment, different dominant enterprises lead their respective supply chains; and (4) the research perspective of the closed-loop supply chain mode selection is relatively single, most of them are based on the pricing strategy and profit distribution strategy, and there are few studies on closed-loop supply chain mode selection from the perspective of logistics mode.

In the current recycling and remanufacturing cycle system, the forward supply chain is usually still dominated by manufacturers, while the backward supply chain is dominated by retailers who control recycling channels [26, 27]. Based on this, this paper mainly studies the forward closed-loop supply chain structure dominated by manufacturers and the backward closed-loop supply chain structure dominated by retailers. Based on the profit distribution mechanism, this paper discusses the logistics mode selection of manufacturers and retailers in the mixed dominant closed-loop supply chain. The research framework is as follows: Section 2 analyzes the closed-loop supply chain model and puts forward basic assumptions. In Section 3, the game model of the closed-loop supply chain model is constructed and the equilibrium solution is obtained.  Section 4 is a comparative analysis of the closed-loop supply chain under different logistics modes. In Section 5, numerical examples are used to verify the research results.

## 2. Model Assumption

**Figure 1:**
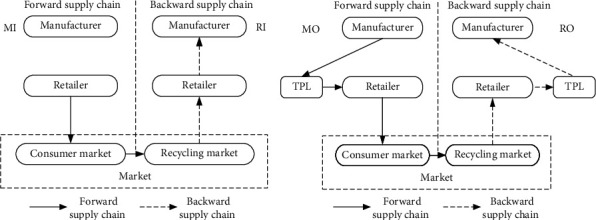
(a) Closed-loop supply chain structure under the logistics self-supporting mode. (b) Closed-loop supply chain structure under the logistics outsourcing mode.

Based on a single sales channel and a single recycling channel, this paper studies the more reasonable logistics mode selection in the closed-loop supply chain. In the forward supply chain, the manufacturer sells the product to retailers, who then provide the product for consumers. In the backward supply chain, recycling retailers recycle the waste products and finally deliver them to the manufacturer for remanufacturing. In the whole closed-loop supply chain process, logistics outsourcing is mainly provided by the third-party logistics enterprises or self-supporting logistics mode. The closed-loop supply chain structure under different logistics modes studied in this paper is shown in Figure 1.

In Figure 1, the manufacturer acts as the front end of the forward supply chain and the end of the reverse supply chain, the consumer market and the recycling market belong to the intermediate links, and the retailer acts as the channel of the forward supply chain and the reverse supply chain. The logistics circulation link between manufacturers and retailers in Figure 1(a) relies on the logistics team of the core enterprise in the supply chain; the logistics circulation link between manufacturers and retailers in Figure 1(b) relies on the third party outside the supply chain. Different logistics operation modes have a great impact on the decision-making of the entire supply chain. On this basis, this paper will further explore which decision-making scheme should be selected by supply chain enterprises under different logistics operation modes to make the overall supply chain operation optimal:

1. The closed-loop supply chain studied in this paper is composed of a single manufacturer, a single retailer, and a single third-party logistics service provider (if logistics outsourcing is selected); it only considers a circulated closed-loop supply chain constituted by single manufacturing, sales forward supply chain and single recovery, and remanufacturing reverse supply chain.
2. As retailers and manufacturers have different statuses in the closed-loop supply chain, core enterprises are usually the leading party in the supply chain. Therefore, this paper sets two situations: manufacturers dominate in a forward supply chain, and retailers dominate in a backward supply chain; retailers dominate in a forward supply chain, and manufacturers dominate in a backward supply chain.
3. Assuming that the demand of the consumer market is determined, the market demand is a linear subtraction function of demand price. *Q* = *α* − *β*p_*r*_, where *α* is the maximum possible demand of the market, i.e., the market size, and *β*＞0 is the price sensitivity coefficient; the quantity of recycled products is $\bar{Q}$, and the expression is $\bar{Q}=k+\gamma h_{r}$, where *k*(*k*＞0) represents the number of products recycled by consumers' environmental awareness [28].
4. When the closed-loop supply chain chooses self-supporting logistics, its unit logistics cost is *θ*. Based on the scale efficiency and professional operation of outsourcing logistics, the forward and backward unit logistics costs of third-party logistics service providers are s*θ*(0 ≤ *s* ≤ 1), respectively, and s*θ* ≤ *p*_*l*_ ≤ *θ*, s*θ* ≤ *p*_*l*_′ ≤ *θ*.
5. As dominant enterprises are at the core of the closed-loop supply chain and have decision-making power, logistics costs are borne by subordinate enterprises in both the forward and backward supply chains. Whether the manufacturer dominates the supply chain or the retailer dominates the supply chain, manufacturers, retailers, and third-party logistics service providers (under logistics outsourcing) in the supply chain will choose decisions that are in line with their own interests.

According to the above assumptions, the profits of manufacturers, retailers, and third-party logistics service providers under the logistics outsourcing mode are as follows:

(1) $$\begin{array}{c} \pi_{m}=\pi_{m1}+\pi_{m2}=\left(p_{m}-c_{m}\right)Q+\left(\Delta -\omega -p_{l}^{\prime }\right)\bar{Q}\text{,}\\ \pi_{l}=\pi_{l1}+\pi_{l2}=\left(p_{l}-s\theta \right)Q+\left(p_{l}^{\prime }-s\theta \right)\bar{Q}\text{,}\\ \pi_{r}=\pi_{r1}+\pi_{r2}=\left(p_{r}-p_{m}-p_{l}\right)Q+\left(\omega -h_{r}\right)\bar{Q}\text{.} \end{array}$$

Under the self-supporting logistics mode, the profits of manufacturers and retailers are as follows:

(2) $$\begin{array}{c} \pi_{m}=\pi_{m1}+\pi_{m2}=\left(p_{m}-c_{m}\right)Q+\left(\Delta -\omega -\theta \right)\bar{Q,}\\ \pi_{r}=\pi_{r1}+\pi_{r2}=\left(p_{r}-p_{m}-\theta \right)Q+\left(\omega -h_{r}\right)\bar{Q}\text{.} \end{array}$$

## 3. Model Building

### 3.1. Game Model and Equilibrium Solution of Logistics Outsourcing Closed-Loop Supply Chain

#### 3.1.1. Forward Supply Chain Game Equilibrium Dominated by Manufacturers (*MO*)

(3) $$\begin{array}{c} \max_{p_{m}}\pi_{m1}=\left(p_{m}-c_{m}\right)Q, \end{array}$$

(4) $$\begin{array}{c} s.t.\;\max_{p_{l}}\pi_{l1}=\left(p_{l}-s\theta \right)Q, \end{array}$$

(5) $$\begin{array}{c} s.t.\;\max_{p_{r}}\pi_{r1}=\left(p_{r}-p_{m}-p_{l}\right)Q\text{.} \end{array}$$

Based on the reverse solving method of Stackelberg game, in the forward supply chain system dominated by manufacturers, first of all, manufacturers predict market demand and then determine their production plan and wholesale price. Secondly, the third-party logistics service providers determine the price of logistics services according to product transportation scale. Finally, sellers determine the product's sales price according to the wholesale and logistics service price. Therefore, it is easy to get the following statement (see Table 1 for equilibrium solutions).

**Proposition 1 .**

Under the logistics outsourcing mode, when the forward supply chain reaches game equilibrium under the leadership of the manufacturer, the wholesale price of the manufacturer, the sales price of the retailer, the service price of the third-party logistics service provider, the market demand, the manufacturer's profit, the retailer's profit, and the logistics service provider's profit are as follows:

(6) $$\begin{array}{c} p_{m}=\frac{\alpha +\beta c_{m}-\beta s\theta }{2\beta },\\ p_{l}=\frac{\alpha +3\beta s\theta -\beta c_{m}}{4\beta },\\ p_{r}=\frac{7\alpha +\beta s\theta +\beta c_{m}}{8\beta },\\ Q=\frac{\alpha -\beta s\theta -\beta c_{m}}{8},\\ \pi_{m1}=\frac{{\left(\alpha -\beta s\theta -\beta c_{m}\right)}^{2}}{16\beta },\\ \pi_{r1}=\frac{{\left(\alpha -\beta s\theta -\beta c_{m}\right)}^{2}}{64\beta },\\ \pi_{l1}=\frac{{\left(\alpha -\beta s\theta -\beta c_{m}\right)}^{2}}{32\beta }\text{.} \end{array}$$

**Proof**

The reverse solution is carried out according to Stackelberg game theory. The retailer's decision should be optimized first. Because *d*^2^*π*_*r*1_/*dp*_*r*_^2^=−2*β*＜0, the first-order condition *dπ*_*r*1_/*dp*_*r*_=0 is the retailer's optimal decision. Thus, according to *dπ*_*r*1_/*dp*_*r*_=0, *p*_*r*_(*p*_*m*_, *p*_*l*_)=*α*+*βp*_*m*_+*βp*_*l*_/2*β*. After substituting *p*_*r*_(*p*_*m*_, *p*_*l*_) into (4), the first-order condition is *p*_*l*_(*p*_*m*_)=*α* − *βp*_*m*_+*βsθ*/2*β*. After substituting*p*_*l*_(*p*_*m*_)into *p*_*r*_(*p*_*m*_, *p*_*l*_), *p*_*r*_(*p*_*m*_)=3*α*+*βp*_*m*_+*βsθ*/4*β* can be obtained, and then *p*_*r*_=7*α*+*βsθ*+*βc*_*m*_/8*β* and *p*_*l*_=*α*+3*βsθ* − *βc*_*m*_/4*β*. After substituting *p*_*m*_,*p*_*r*_, *p*_*l*_ into (3), (4), and (5), the profits of manufacturers, third-party logistics service providers, and retailers can be obtained as follows: *π*_*m*1_=(*α* − *βsθ* − *βc*_*m*_)^2^/16*β*, *π*_*l*1_=(*α* − *βsθ* − *βc*_*m*_)^2^/32*β*, and *π*_*r*1_=(*α* − *βsθ* − *βc*_*m*_)^2^/64*β*.

The first-order derivative of the optimal price to the logistics cost *θ* is conducted to obtain the following results: *∂p*_*m*_/*∂θ*=−(*s*/2)＜0, *∂p*_*l*_/*∂θ*=(3*s*/4)＞0, and *∂p*_*r*_/*∂θ*=(*s*/8)＞0. This shows that the optimal prices *p*_*l*_and *p*_*r*_increase as the logistic cost *θ* increases and decrease as the logistic cost *θ* increases. According to *∂Q*/*∂θ*=−(*βs*/8) < 0, it can be known that *Q* increases with the increase of logistics cost *θ*. In addition, we also know *∂π*_*m*1_/*∂θ*=−(*s*/8)(*α* − *βsθ* − *βc*_*m*_),*∂π*_*r*1_/*∂θ*=−(*s*/32)(*α* − *βsθ* − *βc*_*m*_), and *∂π*_*l*1_/*∂θ*=−(*s*/16)(*α* − *βsθ* − *βc*_*m*_). As *α* − *βsθ* − *βc*_*m*_＞0, it can be known that *∂π*_*m*1_/*∂θ*,*∂π*_*r*1_/*∂θ*, and *∂π*_*l*1_/*∂θ* are less than 0. This indicates optimal profits *π*_*m*1_,*π*_*r*1_, and *π*_*l*1_ decrease as the logistics cost *θ* increases. Besides, the trend of change of *p*_*m*_, *p*_*r*_, *p*_*l*_, *Q*, *π*_*m*1_, *π*_*r*1_, and *π*_*l*1_ is related to the value of S, which is caused by logistics outsourcing.

According to Proposition 1, it can be found that, in the forward supply chain, when the logistics cost S of the third-party logistics service provider is high, the wholesale price of the manufacturer will decrease, the price of the retailer will increase, and the product demand will decrease. This is because in the forward supply chain, the manufacturer is in a dominant position, and retailers of affiliate companies need to bear the cost of logistics. To let retailers gain profit margins, the manufacturer will lower wholesale prices. At the same time, retailers will increase retail prices and transfer costs to consumers in order to deal with high logistics costs, which will produce a vicious cycle. Therefore, consumers will buy fewer products, resulting in a lower quantity of product demand. Finally, the profits of the manufacturers and retailers will decrease. Thus, manufacturers and retailers need to strengthen cooperation with third-party logistics service providers to reduce logistics costs as much as possible and gain more profits.

#### 3.1.2. Backward Retailer-Dominated Supply Chain Game Equilibrium (*RO*)

(7) $$\begin{array}{c} \max_{h_{r}}\pi_{r2}=\left(\omega -h_{r}\right)\bar{Q} \end{array}$$

(8) $$\begin{array}{c} s.t.\;\max_{p_{l}^{\prime }}\pi_{l2}\left(p_{l}^{\prime }-s\theta \right)\bar{Q} \end{array}$$

(9) $$\begin{array}{c} s.t.\;\max_{\omega }\pi_{m2}=\left(\Delta -\omega -p_{l}^{\prime }\right)\bar{Q}\text{.} \end{array}$$

Based on the reverse solving method of the Stackelberg game, in a backward retailer-dominated supply chain system, the seller first determines the recycling price according to the recycling amount of waste products. Then, the third-party logistics service providers determine the price of logistics according to the number of waste products. Finally, the manufacturer determines the transfer price according to the recycling price and service price. Meanwhile, when the manufacturer brings recycled products to the market at the same wholesale price, it is easy to get the following proposition (see Table 1 for equilibrium solutions).

**Proposition 2 .**

Under the logistics outsourcing mode, when the backward supply chain reaches game equilibrium under the dominance of retailers, the manufacturer's transfer price, retailers' recovery price, third-party logistics service providers' service price, market demand, manufacturer's profit, retailers' profit, and logistics service providers' profit are as follows:

(10) $$\begin{array}{c} \begin{array}{c} \omega =\frac{-3k+5\gamma \Delta -5\gamma s\theta }{8\gamma },\\ h_{r}=\frac{-7k+\gamma \Delta -\gamma s\theta }{8\gamma },\\ p_{l}^{\prime }=\frac{k+3\gamma s\theta +\gamma \Delta }{4\gamma }, \end{array}\\ \begin{array}{c} \bar{Q}=\frac{k+\gamma \Delta -\gamma s\theta }{8},\\ \pi_{m2}=\frac{{\left(k+\gamma \Delta -\gamma s\theta \right)}^{2}}{64\gamma },\\ \begin{array}{c} \pi_{r2}=\frac{{\left(k+\gamma \Delta -\gamma s\theta \right)}^{2}}{16\gamma },\\ \pi_{l2}=\frac{{\left(k+\gamma \Delta -\gamma s\theta \right)}^{2}}{32\gamma }. \end{array} \end{array} \end{array}$$

**Proof**

The reverse solution is carried out according to Stackelberg game theory, and unit expected revenue of the retailer is set as *f*, then *ω*=*h*_*r*_+*f*. *ω*=*h*_*r*_+*f* is substituted into (9). The first step is to make the manufacturer's decision optimal, because *d*^2^*π*_*m*2_/*dω*^2^=−2*λ*＜0, as the first order is 0, *ω*(*f*, *p*_*l*_′)=−*k*+*γf*+*γ*Δ − *γp*_*l*_′/2*γ*. After substituting *ω*(*f*, *p*_*l*_′)into *ω*=*h*_*r*_+*f*,*h*_*r*_(*f*, *p*_*l*_′)=−*k* − *γf*+*γ*Δ − *γp*_*l*_′/2*γ* can be obtained, and then it is substituted into (8), *p*_*l*_′(*f*)=*k* − *γf*+*γ*Δ+*γsθ*/2*γ*can be obtained by the first-order condition. Substituting *p*_*l*_′(*f*) and *h*_*r*_(*f*, *p*_*l*_′) into (7), *p*_*r*_(*p*_*m*_)=3*α*+*βp*_*m*_+*βsθ*/4*β* can be obtained. Substituting *p*_*r*_(*p*_*m*_) into (9), the first-order condition is 0, and then *f*=*k*+*γ*Δ − *γsθ*/2*γ*, so *ω*=−3*k*+5*γ*Δ − 5*γsθ*/8*γ*, *h*_*r*_=−7*k*+*γ*Δ − *γsθ*/8*γ*, and *p*_*l*_′=*k*+3*γsθ*+*γ*Δ/4*γ*. By substituting *ω*, *h*_*r*_, *p*_*l*_′ into (7), (8), and (9), the profits of manufacturers, third-party logistics service providers, and retailers can be obtained: *π*_*m*2_=(*k*+*γ*Δ − *γsθ*)^2^/64*γ*, *π*_*l*2_=(*k*+*γ*Δ − *γsθ*)^2^/32*γ*, and *π*_*r*2_=(*k*+*γ*Δ − *γsθ*)^2^/16*γ*.

The first-order derivative of the optimal price to the logistics cost *θ* can be obtained: *∂ω*/*∂θ*=−(5*s*/8)＜0, *∂p*_*l*_′/*∂θ*=3*s*/4＞0, and *∂h*_*r*_/*∂θ*=−(*s*/8)＜0. This indicates that the optimal price *p*_*l*_ increases with the increase of logistics cost *θ*, while *ω* and *h*_*r*_decrease with the increase of logistics cost *θ*. From the equation $\partial \bar{Q}/\partial \theta =-\left(\gamma s/8\right)＜0$, it can be seen that *Q* increases with the increase of logistics cost *θ*. Besides, *∂π*_*m*2_/*∂θ*=−(*s*/32)(*k*+*γ*Δ − *γsθ*), *∂π*_*r*2_/*∂θ*=−(*s*/8)(*k*+*γ*Δ − *γsθ*), and *∂π*_*l*2_/*∂θ*=−(*s*/16)(*k*+*γ*Δ − *γsθ*). As *k*+*γ*Δ − *γsθ*＞0, *∂π*_*m*2_/*∂θ*, *∂π*_*r*2_/*∂θ*, and *∂π*_*l*2_/*∂θ* are less than 0. This indicates that the optimal profits *π*_*m*2_,*π*_*r*2_, and *π*_*l*2_decrease as the logistics cost *θ* increases, while the variation tendency of *ω*, *h*_*r*_, $\bar{Q}$, *π*_*m*2_, *π*_*r*2_, and *π*_*l*2_is related to S, which is correlated with logistics outsourcing.

According to Proposition 2, it can be found that, in the backward supply chain, the larger the logistics cost(S) is, the larger the transfer price of the manufacturer is, and the higher the recycling price of the retailer is, but the lower the number of recycled waste products will be. This is because the backward supply chain is dominated by retailers and the manufacturers bear the cost of logistics outsourcing; to obtain certain profits from remanufacturing, manufacturers reduce the transfer price. At this time, the profits obtained by the manufacturers from retailers will also reduce, thus resulting in a declined recycling price and eventually leading to a decline in enterprises' profits. Therefore, backward recycling channels also need to take corresponding measures to reduce logistics costs.

### 3.2. Game Model and Equilibrium Solution of Logistics Self-Supporting Closed-Loop Supply Chain

#### 3.2.1. Forward Supply Chain Game Equilibrium Dominated by Manufacturers (*MI*)

(11) $$\begin{array}{c} \max_{p_{m}}\pi_{m1}=\left(p_{m}-c_{m}\right)Q, \end{array}$$

(12) $$\begin{array}{c} s.t.\;\max_{p_{r}}\pi_{r1}=\left(p_{r}-p_{m}-\theta \right)Q\text{.} \end{array}$$

According to the reverse solution method of the Stackelberg game, in the forward manufacturer-dominated supply chain system, the manufacturer first predicts the market demand to determine its production plan and wholesale price, and the seller decides the sales price of the product according to the wholesale price; thus, the following proposition can be easily obtained (see Table 1 for equilibrium solutions).

**Proposition 3 .**

Under the logistics self-supporting mode, when the forward supply chain reaches game equilibrium under the leadership of the manufacturer, the wholesale price of the manufacturer, the selling price of the retailer, the market demand, the manufacturer's profit, and the retailer's profit are as follows:

(13) $$\begin{array}{c} \begin{array}{c} p_{m}=\frac{\alpha -\beta c_{m}+\beta \theta }{2\beta },\\ p_{r}=\frac{3\alpha +\beta c_{m}+\beta \theta }{4\beta },\\ Q=\frac{\alpha -\beta c_{m}-\beta \theta }{4}, \end{array}\\ \begin{array}{c} \pi_{m1}=\frac{{\left(\alpha -\beta c_{m}-\beta \theta \right)}^{2}}{8\beta },\\ \pi_{r1}=\frac{{\left(\alpha -\beta c_{m}-\beta \theta \right)}^{2}}{16\beta }. \end{array} \end{array}$$

**Proof**

According to the reverse solution, the first order of (12) is 0, so *p*_*r*_(*p*_*m*_)=*α*+*βp*_*m*_/2*β*. Substituting *p*_*r*_(*p*_*m*_) into (11), as the first order is 0, *p*_*m*_=*α*+*βc*_*m*_ − *βθ*/2*β*, and then *p*_*r*_=3*α*+*βc*_*m*_+*βθ*/4*β*, *Q*=*α* − *βc*_*m*_ − *βθ*/4, *π*_*m*1_=(*α* − *βc*_*m*_ − *βθ*)^2^/8*β*, and *π*_*r*1_=(*α* − *βc*_*m*_ − *βθ*)^2^/16*β*.

The first-order derivative of the optimal price to the logistics cost *θ* can be obtained: *∂p*_*m*_/*∂θ*=−(1/2)＜0, *∂p*_*r*_/*∂θ*=(1/4)＞0. This indicates that the optimal price *p*_*r*_increases with the increase of logistics cost *θ*, while *p*_*m*_decreases with the increase of logistics cost *θ*. From *∂Q*/*∂θ*=−(*β*/4)＜0, it can be seen that *Q* decreases with the increase of logistics cost *θ*. Besides, *∂π*_*m*1_/*∂θ*=−(1/4)(*α* − *βθ* − *βc*_*m*_)and *∂π*_*r*1_/*∂θ*=−(1/8)(*α* − *βθ* − *βc*_*m*_). As *α* − *βθ* − *βc*_*m*_＞0, both *∂π*_*m*1_/*∂θ* and *∂π*_*r*1_/*∂θ* are less than 0, which indicates that the optimal profits *π*_*m*1_ and *π*_*r*1_decrease as the logistics cost *θ* increases.

According to Proposition 3, it can be found that, in the forward supply chain, when the logistics cost is higher, the wholesale price and sale price will rise, and the demand of the market can also be reduced accordingly, eventually resulting in a decrease of profits of retailers and manufacturers. Therefore, enterprises must consider the impact of logistics cost on their operation when carrying out self-supporting logistics and then use various management techniques and methods to reduce logistics cost.

#### 3.2.2. Backward Retailer-Dominated Supply Chain Game Equilibrium (*RI*)

(14) $$\begin{array}{c} \max_{h_{r}}\pi_{r2}=\left(\omega -h_{r}\right)\bar{Q}, \end{array}$$

(15) $$\begin{array}{c} s.t.\;\max_{\omega }\pi_{m2}=\left(\Delta -\omega -\theta \right)\bar{Q}\text{.} \end{array}$$

According to the reverse solution method of the Stackelberg game, in the backward retailer-dominated supply chain system, the retailer firstly determines the recycling price according to the recycling amount of waste products, and the manufacturer determines the transfer price according to the recycling price, and then the manufacturer flows the recycled products into the market at the same wholesale price; thus, the following proposition can be easily obtained (see Table 1 for equilibrium solutions).

**Proposition 4 .**

Under the self-supporting logistics mode, when the backward supply chain achieves game equilibrium under the dominance of retailers, the manufacturer's transfer price, retailer's recovery price, market demand, manufacturer's profit, and retailer's profit are as follows:*ω*=−*k* − 3*γθ*+3*γ*Δ/4*γ*, *h*_*r*_=−3*k*+*γ*Δ − *γθ*/4*γ*, $\bar{Q}=k+\gamma \Delta -\gamma \theta /4$, *π*_*m*2_=(*k*+*γ*Δ − *γθ*)^2^/16*γ*, and *π*_*r*2_=(*k*+*γ*Δ − *γθ*)^2^/8*γ*.

**Proof**

Reverse solving, assume that the unit revenue expected by the retailer is *f*, then *h*_*r*_=*ω* − *f*. Then, it is substituted into Equation (152), and according to the condition that the first order is 0, *ω*(*f*)=−*k*+*γf* − *γθ*+*γ*Δ/2*γ* and *h*_*r*_(*f*)=−*k* − *γf*+*γ*Δ − *γθ*/2*γ* can be obtained. After substituting*h*_*r*_(*f*) into (14), *f*=*k*+*γ*Δ − *γθ*/2*r* can be obtained. So, *ω*=−*k* − 3*γθ*+3*γ*Δ/4*γ*, *h*_*r*_=−3*k*+*γ*Δ − *γθ*/4*γ*, $\bar{Q}=k+\gamma \Delta -\gamma \theta /4$, *π*_*m*2_=(*k*+*γ*Δ − *γθ*)^2^/16*γ*, and *π*_*r*2_=(*k*+*γ*Δ − *γθ*)^2^/8*γ*.

The first-order derivative of the optimal price to the logistics cost *θ* is obtained: *∂ω*/*∂θ*=−(3/4)＜0, *∂h*_*r*_/*∂θ*=−(1/4)＜0. This indicates that the optimal prices *ω*and *h*_*r*_decrease as the logistics cost *θ* increases. From $\partial \bar{Q}/\partial \theta =-\left(\gamma /4\right)＜0$, it can be seen that $\bar{Q}$ increases as the logistics cost *θ* increases. Besides, the following equations can be obtained: *∂π*_*m*2_/*∂θ*=−(1/8)(*k*+*γ*Δ − *γθ*) and *∂π*_*r*2_/*∂θ*=−(1/4)(*k*+*γ*Δ − *γθ*). As *k*+*γ*Δ − *γθ*＞0, it can be known that *∂π*_*m*2_/*∂θ* and *∂π*_*r*2_/*∂θ* are less than 0, which indicates that the optimal profits *π*_*m*2_ and *π*_*r*2_decrease as the logistics cost *θ* increases.

According to Proposition 4, it can be found that, in the backward supply chain, when the transfer price and recycling amount of waste products are smaller, the profit of enterprises will decrease with the increase of logistics cost. Therefore, in the logistics self-supporting closed-loop supply chain, manufacturers and distributors should strengthen cooperation to reduce logistics costs. Meanwhile, the higher the cost saving of remanufacturing is, the larger the transfer price and recovery price will be, and the greater the recycling amount will be. Therefore, the manufacturers of recycling and reprocessing should improve the technical level and increase the cost saving of remanufacturing to improve their profits.

## 4. Comparative Analysis of Closed-Loop Supply Chain under Different Logistics Modes

From the perspective of social and economic value, selecting an appropriate logistics mode can improve the profits of the closed-loop supply chain. For the convenience of description and expression, in this paper, *H*,*T*, $\bar{H}$, and $\bar{T}$ are introduced as replacement variables to compare the profits of manufacturers and retailers based on different logistics services. *H*=*α* − *βsθ* − *βc*_*m*_, *T*=k+*γ*(Δ − *sθ*), $\bar{H}=\alpha -\beta \theta -\beta c_{m}$, and $\bar{T}=k+\gamma \left(\Delta -\theta \right)$. As 0 ≤ *s* ≤ 1, $H\geq \bar{H}$ and $T\geq \bar{T}$. Then, the following conclusions are drawn.

**Conclusion 1 .**

In the forward supply chain, when $H/\bar{H}\geq 2$, the product demand of the market under logistics outsourcing is greater than that under self-supporting logistics; when $1＜H/\bar{H}＜2$, the product demand of the market under the logistics self-supporting mode is greater than that under the logistics outsourcing mode. In the backward supply chain, when $T/\bar{T}\geq 2$, the product demand of the market under the logistics outsourcing mode is greater than that under the self-supporting logistics mode; when $1＜T/\bar{T}＜2$, the product demand of the market under the self-supporting logistics mode is greater than that under the logistics outsourcing mode.

**Proof**

As *Q*^*MO*^ − *Q*^*MI*^=*H*/8 − *H*/4, when *Q*_*m*1_^*MO*^ ≥ *Q*_*m*1_^*MI*^, $H/\bar{H}\geq 2$. On the contrary, when *Q*_*m*1_^*MO*^＜*Q*_*m*1_^*MI*^, $H/\bar{H}＜2$, and $H\geq \bar{H}$, then $1＜H/\bar{H}$, so $1＜H/\bar{H}＜2$. Similarly, ${\bar{Q}}^{RO}-{\bar{Q}}^{RI}=T/8-T/4$, when ${\bar{Q}}^{RO}\geq {\bar{Q}}^{RI}$, $T/\bar{T}\geq 2$ and ${\bar{Q}}^{RO}＜{\bar{Q}}^{RI}$, $1＜T/\bar{T}＜2$.

**Conclusion 2 .**

In the forward supply chain, when $1\leq H/\bar{H}＜\sqrt{2}$, the profits of manufacturers and retailers when they choose self-supporting logistics are greater than those when they choose outsourcing logistics; when $\sqrt{2}\leq H/\bar{H}＜2$, the manufacturer chooses logistics outsourcing, whose profit is greater than that of logistics self-supporting, while the retailer chooses logistics self-supporting, whose profit is higher than that of logistics outsourcing. When $H/\bar{H}\geq 2$, the profits of manufacturers and retailers choosing logistics outsourcing are greater than those choosing logistics self-operating.

**Proof**

As $\pi_{m1}^{MO}-\pi_{m1}^{MI}=H^{2}/16\beta -{\bar{H}}^{2}/8\beta$, $\pi_{r1}^{MO}-\pi_{r1}^{MI}=H^{2}/64\beta -{\bar{H}}^{2}/16\beta$, when *π*_*m*1_^*MO*^ ≥ *π*_*m*1_^*MI*^, $H/\bar{H}\geq \sqrt{2}$, and when *π*_*r*1_^*MO*^ ≥ *π*_*r*1_^*MI*^, $H/\bar{H}\geq 2$. On the contrary, when *π*_*m*1_^*MO*^＜*π*_*m*1_^*MI*^, *π*_*m*1_^*MO*^＜*π*_*m*1_^*MI*^, $1\leq H/\bar{H}＜\sqrt{2}$ and $1\leq H/\bar{H}＜2$ ($H\geq \bar{H}$).

**Conclusion 3 .**

In the backward supply chain, when $1\leq T/\bar{T}＜\sqrt{2}$, the profits of manufacturers and retailers when they choose self-supporting logistics are greater than those when they choose outsourcing logistics. When $\sqrt{2}\leq T/\bar{T}＜2$, the profit of the manufacturer when choosing self-supporting logistics is greater than that when choosing logistics outsourcing, but the retailer has a high profit when choosing logistics outsourcing. When $T/\bar{T}\geq 2$, the profit of manufacturers and retailers under the logistics outsourcing mode is greater than that under the logistics self-supporting mode.

**Proof**

As $\pi_{m2}^{RO}-\pi_{m2}^{RI}=T^{2}/64\beta -{\bar{T}}^{2}/16\beta$, $\pi_{r2}^{RO}-\pi_{r2}^{RI}=T^{2}/16\beta -{\bar{T}}^{2}/8\beta$, when *π*_*m*2_^*RO*^ ≥ *π*_*m*2_^*RI*^, $T/\bar{T}\geq 2$, and when *π*_*r*2_^*RO*^ ≥ *π*_*r*2_^*RI*^, $T/\bar{T}\geq \sqrt{2}$. On the contrary, when *π*_*m*2_^*RO*^＜*π*_*m*2_^*RI*^, *π*_*r*2_^*RO*^＜*π*_*r*2_^*RI*^, $1\leq T/\bar{T}＜2$ and $1\leq T/\bar{T}＜\sqrt{2}$ ($T\geq \bar{T}$).

## 5. Numerical Simulation

The logistics mode of the closed-loop supply chain is affected by sales market scale *α*, recycling market base amount *k*, consumer price sensitive coefficients *β* and *γ*, and outsourcing logistics cost ratio *s*, but the fundamental starting point of choosing logistics outsourcing or self-supporting is to reduce logistics cost *θ* and thereby improve the profits of enterprises. Therefore, this paper starts from the perspective of logistics cost *θ*, and the decision-making of the closed-loop supply chain is explored based on the proportion *s* of self-operated logistics and outsourcing logistics costs. The optimal equilibrium solutions under different closed-loop supply chain modes are obtained through numerical simulation, and then the analysis and comparison are carried out.

**Figure 2:**
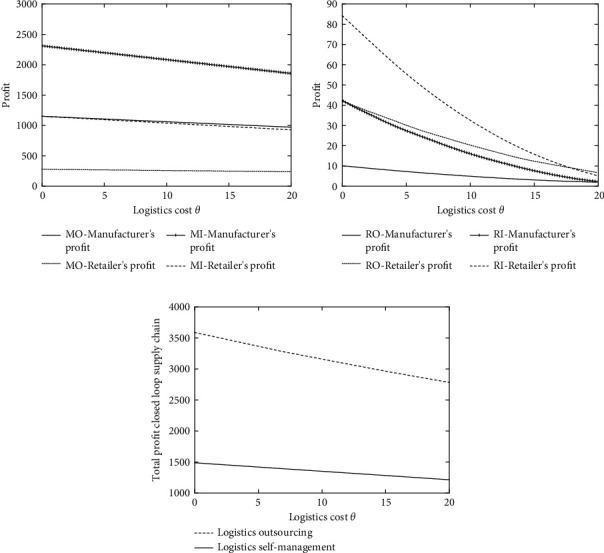
(a) Profit comparison between manufacturers and retailers in the reverse supply chain under different logistics modes (*s* = 0.8). (b) Profit comparison between manufacturers and retailers in the reverse supply chain under different logistics modes (*s* = 0.8). (c) Comparison of total profit of the closed-loop supply chain under different logistics modes (*s* = 0.8).

**Figure 3:**
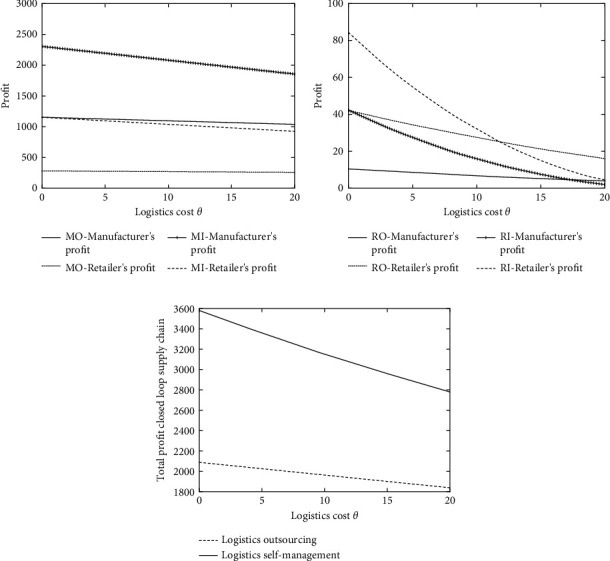
(a) Profit comparison between manufacturers and retailers in the reverse supply chain under different logistics modes (*s* = 0.5). (b) Profit comparison between manufacturers and retailers in the reverse supply chain under different logistics modes (*s* = 0.5). (c) Comparison of total profit of the closed-loop supply chain under different logistics modes (*s* = 0.5).

**Figure 4:**
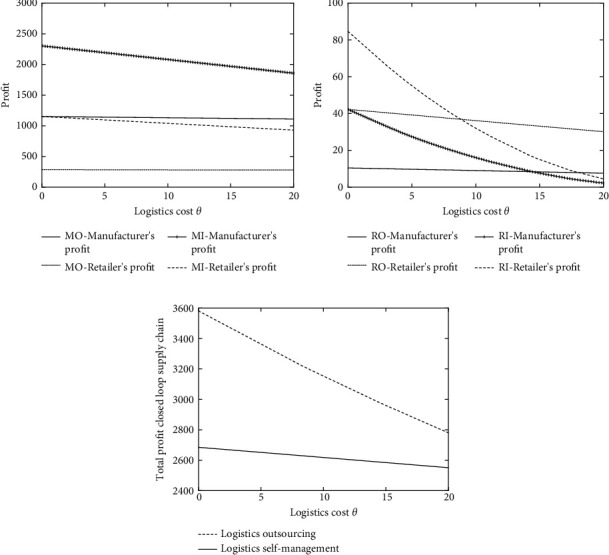
(a) Profit comparison between manufacturers and retailers in the reverse supply chain under different logistics modes (*s* = 0.2). (b) Profit comparison between manufacturers and retailers in the reverse supply chain under different logistics modes (*s* = 0.2). (c) Comparison of total profit of the closed-loop supply chain under different logistics modes (*s* = 0.2).

To simplify the model and facilitate the analysis, the corresponding parameters are assigned as follows: the marginal cost per unit of the manufacturer's production is *c*_*m*_=8 and the marginal cost per unit of manufacturer remanufacturing is *c*_*m*_′=2, so the cost saved by remanufacturing is Δ=c_*m*_ − *c*_*m*_′=6, *α*=100, *k*=20, *β*=0.5, *γ*=1, *Q*=100 − 0.5*p*_*r*_, and $\bar{Q}=20+h_{r}$. In this paper, the values when *s*=0.8, *s*=0.5, and *s*=0.2 are selected and the analysis results are shown in Figures 2–4.

It can be seen from Figures 2(a), 3(a), and 4(a) that, in the forward closed-loop supply chain, as the logistics cost *θ* increases, the profits of manufacturers and retailers under different logistics modes will gradually decrease. When the logistics cost is very low, the profits of manufacturers and retailers when selecting the logistics outsourcing mode are lower than those when selecting the logistics self-supporting mode, and manufacturers and retailers are more willing to accept the self-supporting logistics mode. However, as the logistics cost *θ* rises, although the profits of manufacturers and retailers choosing logistics outsourcing and logistics self-supporting modes all decline, the decline speed of the profits under the logistics outsourcing mode is far less than that under the logistics self-supporting mode, which means that when the logistics cost is very high, the advantages of logistics outsourcing are very outstanding, so at this time, it is better to choose logistics outsourcing. Similarly, in the backward supply chain, according to Figures 2(b), 3(b), and 4(b), it can be known that, under different values of *s*, when the logistics cost rises slowly, the economic benefits of logistics outsourcing become more obvious, and enterprises are more willing to choose the outsourcing logistics mode.

This is consistent with the reality. Generally, supply chain companies are in the period of business growth. Due to the small business volume, the logistics and transportation of goods between manufacturers and retailers will choose the self-operated logistics mode, which is highly flexible and convenient. However, as the business volume of the enterprise increases, the circulation pressure borne by the own logistics team increases, the original logistics investment cannot meet the existing business volume, and the logistics cost will continue to rise. At this time, logistics outsourcing can effectively relieve the operating pressure of enterprises. At the same time, allowing a professional logistics team to operate will improve efficiency, which can effectively reduce logistics costs and achieve a win-win situation.

According to Figures 2(c), 3(c), and 4(c), it can be found that the total profit of the closed-loop supply chain decreases with the increase of logistics cost, which is consistent with the willingness of enterprises to reduce logistics cost vigorously in reality. In the comparison of logistics outsourcing and logistics self-supporting, it can be found when the logistics cost is low, the total profit of the closed-loop supply chain when selecting logistics outsourcing is far lower than that when selecting logistics self-supporting, which suggests that, under the low logistics cost, logistics outsourcing cannot bring enough profits to enterprises in the supply chain, and on the contrary, self-supporting logistics can make enterprises gain more profits. As can be seen from the straight downward trend in the figure, when the logistics cost is high enough, no matter what the value of *s* is, the profit of logistics outsourcing will gradually exceed that of self-supporting logistics, and at this time, the professionalism and scale of logistics outsourcing will be reflected.

As a whole, at different levels of *s*, with the increase of logistics cost *θ*, both the forward product demand and the reverse backward product recovery will decrease when the logistics cost increases, regardless of the self-supporting logistics mode or the logistics outsourcing mode. This is because logistics cost, as the main component of enterprise circulation (including recycling) cost, directly affects products' sales (recycling) price, thus further influencing the profits of enterprises. The high cost of self-supporting logistics will increase the burden of manufacturing enterprises or retail enterprises, which will lead to enterprises preferring the logistics outsourcing mode.

For the overall closed-loop supply chain, the level of logistics costs will affect the total profit of the overall supply chain. Pursuing the maximization of the overall profit of the closed-loop supply chain, it is necessary to take into account the profits of each subject in the closed-loop supply chain. At this time, how to control the logistics cost becomes an effective way to expand the profit. When the logistics cost is low, choosing the self-operated logistics mode is beneficial to reducing the total cost of the closed-loop supply chain, thereby increasing the profit of the closed-loop supply chain. Scale and specialization improve the efficiency of the overall closed-loop supply chain, which can also achieve the goal of reducing costs and increasing efficiency in the closed-loop supply chain.

## 6. Conclusion

In this paper, a closed-loop supply chain structure of a single manufacturer and retailer was constructed, in which the forward supply chain is dominated by the manufacturer, while the backward supply chain is dominated by the seller. By considering logistics outsourcing and logistics self-supporting modes simultaneously, the impact of choosing a logistics mode for the supply chain under a hybrid dominant mode on the pricing decisions of manufacturers and retailers was studied. Based on the analysis of market demand, recycling volume, and profits of manufacturers and retailers under the two logistics modes, which logistics mode was more reasonable under different leading enterprises in the forward and backward supply chains was analyzed, and numerical simulation was used to verify the conclusions. The conclusions are as follows:

1. Decision-making when market demand and recovery volume are the largest: In the forward supply chain link, when $H/\bar{H}\geq 2$, logistics outsourcing is superior to logistics self-supporting; when $1＜H/\bar{H}＜2$, logistics self-supporting is better than logistics outsourcing decision. In the backward supply chain, when $T/\bar{T}\geq 2$, logistics outsourcing is superior to logistics self-supporting; when $1＜T/\bar{T}＜2$, logistics self-supporting is better than logistics outsourcing.
2. Manufacturer's optimal decision: In the forward supply chain, when $1\leq H/\bar{H}＜\sqrt{2}$, the manufacturer had a higher profit when choosing a self-supporting logistics strategy; when $H/\bar{H}\geq \sqrt{2}$, the manufacturer had a higher profit when choosing logistics outsourcing. In the backward supply chain, the manufacturer had a higher profit when choosing the logistics outsourcing mode; when $1\leq T/\bar{T}＜2$, the manufacturer had a higher profit when choosing the logistics self-supporting mode.
3. Retailer's optimal decision: In the forward supply chain, when $1\leq H/\bar{H}＜2$, the manufacturer had a higher profit when choosing a self-supporting logistics strategy; when $H/\bar{H}\geq 2$, the manufacturer had a higher profit when choosing logistics outsourcing. In the backward supply chain, when $T/\bar{T}\geq \sqrt{2}$, the manufacturer had a higher profit when choosing the logistics outsourcing mode; when $1\leq T/\bar{T}＜\sqrt{2}$, the manufacturer had a higher profit when choosing the logistics self-supporting mode.

As the decision-making of the forward supply chain and backward supply chain is dominated by different enterprises, the forward supply chain and backward supply chain can be seen as two separate supply chains. The decision is made according to the optimal combination, and the closed-loop supply chain eventually formed can be a closed-loop supply chain outsourcing and whole self-supporting, which also can be said to make the forward supply chain outsourcing and the backward supply chain self-supporting, and the principle is to maximize corporate profits.

Future research can be carried out from the following perspectives: on the one hand, the decision-making problem of the closed-loop supply chain under uncertain market demand should be studied, and at the same time, the choice mode of logistics mode under two-way and dual-channel, namely, two-way sales channel and two-way recovery channel, should be further studied; on the other hand, the closed-loop supply chain constructed by a single manufacturer and a single retailer should be extended to a competitive closed-loop supply chain constituted by multiple manufacturers and multiple retailers, and carrying out an in-depth study on this type of supply chain decision-making will have stronger theoretical and practical significance.

## Figures and Tables

**Table 1:** Equilibrium solutions of closed-loop supply chain under different logistics modes.

	Logistics outsourcing		Logistics self-supporting	
	*MO*	*RO*	*MI*	*RI*
*Q*	*α* − *βsθ* − *βc*_*m*_/8	—	*α* − *βθ* − *βc*_*m*_/4	—
$\bar{Q}$	—	*k*+*γ*Δ − *γsθ*/8	*k*+*γ*Δ − *γθ*/4	*k*+*γ*Δ − *γθ*/4
*π*_*m*_	(*α* − *βsθ* − *βc*_*m*_)^2^/16*β*	(*k*+*γ*Δ − *γsθ*)^2^/64*γ*	(*α* − *βθ* − *βc*_*m*_)^2^/8*β*	(*k*+*γ*Δ − *γθ*)^2^/16*γ*
*π*_*r*_	$\frac{{\left(\alpha -\beta s\theta -\beta c_{m}\right)}^{2}}{64\beta }$	(*k*+*γ*Δ − *γsθ*)^2^/16*γ*	(*α* − *βθ* − *βc*_*m*_)^2^/16*β*	(*k*+*γ*Δ − *γθ*)^2^/8*γ*
*π*_*l*_	$\frac{{\left(\alpha -\beta s\theta -\beta c_{m}\right)}^{2}}{32\beta }$	(*k*+*γ*Δ − *γsθ*)^2^/32*γ*	—	—

## Data Availability

The experimental data used to support the findings of this study are available from the corresponding author upon request.
